# Sexes Are Polymorphisms: Describing and Understanding Sexual Diversity as Phenotypic Variation

**DOI:** 10.1093/icb/icag132

**Published:** 2026-07-28

**Authors:** Jay Jinsing Falk

**Affiliations:** Center for Animal Behavior, Smithsonian Tropical Research Institute, Balboa, Ancón 0843-03092, Republic of Panama; Department of Ecology and Evolution, Princeton University, Princeton, NJ 08540, USA

## Abstract

Recent discussion has emphasized the need for more precise frameworks for describing sex. Sexes are a type of within-species phenotypic variation, and treating them as such offers a valuable perspective across levels of scientific inquiry. I propose that phenotypic polymorphism—the presence of distinct morphs within a species—provides an especially relevant framework for studying sexes as a form of variation. Although sexes are themselves a type of polymorphism, they are rarely described in these terms. Drawing on examples from diverse taxa, I demonstrate that the mechanisms underlying the production and adaptive maintenance of polymorphisms are the same as those that produce sexes. No genetic, developmental, or evolutionary principle distinguishes sexes from other polymorphisms. I further highlight cases in which polymorphisms play as central a role to reproduction as sexes, underscoring how a polymorphism-based approach can capture the diversity of reproductive strategies while improving conceptual frameworks. By treating sexes as a form of phenotypic variation, the polymorphism perspective clarifies that sexes are a population-level trait, and that existing tools for analyzing variation within and across species can be used for a more descriptive and empirical understanding of reproductive types and their evolution.

## Abstract (es)

Discusiones recientes han resaltado la necesidad de marcos más precisos para describir los sexos. Los sexos constituyen un tipo de variación fenotípica intraespecífica y tratarlos como tal ofrece una perspectiva valiosa en todos los niveles de la investigación científica. Propongo que el polimorfismo fenotípico—la presencia de morfos distintos dentro de una especie—proporciona un marco especialmente relevante para estudiar los sexos como una forma de variación. Aunque los sexos son en sí mismos un tipo de polimorfismo, rara vez se describen en estos términos. Utilizando ejemplos de diversos taxones, demuestro que los mecanismos que subyacen a la aparición y el mantenimiento adaptativo de los polimorfismos son los mismos que los que dan lugar a los sexos. No existe ningún principio genético, de desarrollo o evolutivo que distinga a los sexos de otros polimorfismos. Además, destaco los casos en los que los polimorfismos desempeñan un papel tan fundamental en la reproducción como los sexos, lo que resalta cómo un enfoque basado en los polimorfismos puede reflejar la diversidad de estrategias reproductivas mientras se construyen mejores marcos conceptuales. Al considerar los sexos como una forma de variación fenotípica, la perspectiva del polimorfismo deja claro que los sexos son un rasgo a nivel poblacional y que las herramientas existentes para analizar la variación dentro de las especies y entre ellas pueden utilizarse para obtener una comprensión más descriptiva y empírica de los tipos reproductivos y su evolución.

**Cover image:** Long-leaved bluets, *Houstonia longifolia*, are distylous, a polymorphism in which some flowers have long styles and short stamens (pin morph, right two flowers), others have short styles and long stamens (thrum morph, left two flowers). Like sexes, dystylous species have restricted reproduction between morphs of the same type.

## Introduction

There has been recent debate regarding the utility of binary versus more complex models of sexes (Fausto-Sterling 2000; Goymann et al. 2023; McLaughlin et al. 2023; Smiley et al. 2024; Griffiths and Spencer 2025; Eppley et al. 2026). In the binary model, individuals can almost always be classified by classes of gamete size: males produce the smaller class, females the larger class, and hermaphrodites both. Proponents of this definition typically acknowledge that there is extensive variation in the phenotypes classified as male versus female, especially across taxa. This, they argue, is why it is critical to have a clear definition of sexes (i.e., producers of gamete size classes) that can easily be compared across taxa (Roughgarden 2013; Goymann et al. 2023; Griffiths and Spencer 2025). On the other hand, proponents of a multivariate model argue that gamete size classes cannot single-handedly define sexes because they have little bearing on the broader phenotypes of individuals, and fail to reflect the highly complex nature of sexual phenotypes (McLaughlin et al. 2023; Smiley et al. 2024).

These two models of sexes identify a common problem, but emphasize different approaches to describing the nature of complex sexual phenotypes; neither model is functionally appropriate in all contexts (Ritz and DuBois 2025; Warkentin et al. 2026a). The binary model allows for simplified comparison across disparate taxa and independent evolutions of anisogamy, but provides limited predictive power about the evolution of sex-specific traits, especially across broad swaths of the tree of life and independent evolutions of anisogamy (Ah-King 2013; Mokos et al. 2021; Vries and Lehtonen 2023). Furthermore, generalized definitions used to compare sexes across taxa, such as anisogamy, are slippery when applied across eukaryotes (Warkentin et al. 2026a). A multivariate model, on the other hand, complicates cross‐taxonomic comparison by trading categorical clarity for a more complete representation of biological diversity. Both models involve the description of traits, but there is tension in how those traits should be organized, weighted, and compared. Acknowledging these differences shifts attention toward a fundamental challenge: How can the complexity of sexual phenotypes be coherently characterized and compared across taxa in a way that accommodates, rather than collapses, this complexity?

From a very broad perspective, sexes are a type of within-species biological variation. Given that sexes are often treated as discrete categories, it may be particularly useful to view sexes as phenotypic *polymorphisms* (Box 1; Ford 1945; Stehr 1964). Phenotypic polymorphisms are distinct forms within a species, and can be determined by genetic variation, environmental cues, or both (Box 1; Ford 1945). Treating sexes as a type of variation implies that the sexual phenotype is best understood as a population-level distribution of correlated traits rather than as a discrete individual attribute. Sexual phenotypes can be represented as regions of a multidimensional trait space defined by correlated morphological, physiological, and behavioral traits, with the familiar male–female distinction corresponding to clusters of variation in the aggregated sexual phenotype (Warkentin et al. 2026a). Crucially, this places sexes in precisely the same framework as other polymorphic trait complexes studied in evolutionary and organismal biology.

In this paper, I explore a “polymorphism perspective” of sexes—that *sexes are a type of within-species phenotypic variation that often take the form of distinctive morphs*. Variation is the fundamental material of evolution, and the field of evolutionary biology has developed endless methods for measuring, visualizing, and analyzing population variation. Sexes should be subject to this same analytical perspective, yet are often treated as an inherent, static, monolithic, and fundamental aspect of a species’ natural history. Describing sex as what it is—one of many layers of variation that comprise a species—provides a scaffold for viewing sexes within the broader landscape of phenotypic diversity, enabling it to be studied with the same theoretical and empirical tools applied to any other trait.

A polymorphism perspective of sexes acknowledges that sexes are complex and comprised of multiple traits (*sensu* McLaughlin et al. 2023). However, just like any other polymorphism in nature, researchers can describe each morph (or sex) based on a set of defined criteria and boundaries. The practice of defining such criteria allows for an opportunity to compare across taxa with clarity, while also acknowledging the internal complexity of each morph—the suite of covarying traits, distribution outliers, and the variation among individuals that are typically left implicit when sexes are treated as self-evident. A polymorphism perspective complements rather than supplants existing models of sex, offering a vantage shift derived from basic principles of biological variation. Taking such a polymorphism perspective does not eliminate or obviate sex as a biologically relevant concept. Instead, it grounds sexes alongside other types of variation, and encourages specificity. Doing so may encourage crosstalk between fields studying sexes and other polymorphisms, potentially leading to generalized frameworks.

Box 1 What is a polymorphism?

The term “polymorphism” has been used in various contexts by different fields. One of the earliest explicit definitions came from Ford (1945), which I use in this paper: *the occurrence together in the same habitat of two or more distinct forms of a species*. Although later usages of the term by geneticists described polymorphisms as a genetic phenomenon, Ford’s definition was explicitly phenotypic, and included forms derived from both genetic and environmental influences (Falk et al. 2026). Indeed, Ford recognized that “*Sex itself is a true balanced polymorphism, environmentally or genetically controlled*” and provided some discussion on the implications, such as the fact that sexes must be evolutionary balanced near an equal sex ratio.

Exactly what types of variation are considered a polymorphism? Ford’s description is simple: “[Polymorphism] relates not to ‘continuous variation’, such as that falling within a single normal distribution, but to relatively sharply contrasted differences which either do not overlap or else give rise to a bi-modal (or multi-modal) curve.” Therefore, polymorphisms flexibly encompass modal trait distributions that are either overlapping or highly distinct. This early, deliberately broad definition is crucial when describing the myriad sexual traits in any given organism, which may vary in the shape and degree of their modality. All individuals, whether they fall neatly into categorized morphs, are a real and important part of phenotypic distributions.

Although often not explicitly stated, morphs do not typically differ in a single trait but encompass several associated traits resulting in complex correlations. Even the simplest of genetic mechanisms are typically pleiotropic (Chesmore et al. 2018; Mackay and Anholt 2024), causing the correlation or association of multiple phenotypes. Therefore, a common characteristic of polymorphisms is the presence of correlated trait complexity. Though sexes often are quite similar in aspects other than reproductive organs, trait association can take extreme forms in some species, with females and males diverging strongly in multiple aspects of appearance or behavior. Trait association in sexes has been described by some as a “mosaicism” (West-Eberhard 2003a), and can be envisioned as a multi-dimensional trait space (Warkentin et al. 2026a), or as stacked layers of multiple trait distributions (see section: A polymorphic perspective of sexes). Here again, associations may not be strict, and traits often found in one morph can sometimes be expressed in another. Within and between morphs, trait associations and disassociations can occur through plasticity, selection, or non-adaptive evolution (West-Eberhard 2003b). Less common associations between reproductive traits are meaningful rather than anomalous, reflecting the many ways traits can combine rather than a single typical pattern.

When describing “distinct” sexes, it can be tempting to over-categorize. These two attributes of polymorphisms, trait overlap and association flexibility, offer a means to avoid the assumption of highly distinct categorization. The overlap and fluidity of trait association is not only relevant to how we conceptualize sexual phenotypes, but also has important evolutionary consequences—for example, through the development of integrated phenotypes and cross-sexual transfers (West-Eberhard 2003a; Anderson and Falk 2023).

In this paper, I will compare notable examples of sexual and non-sexual polymorphisms from a variety of perspectives. The concept that sexes are a type of within-species variation is not new (Stehr 1964; Ford 1966), but is often passed over quickly without further conceptual development. Here I focus on comparisons between sexes and other types of variation, demonstrating that there is no foundational or qualitative distinction between sex and non-sex polymorphisms, other than being defined as such. First, I explore the genetic and developmental basis of polymorphisms—what are the physical mechanisms by which correlated traits are expressed together? Second, I delve into the maintenance of polymorphism through selection—why are there multiple morphs, rather than one? I finish by discussing some advantages of taking a polymorphism perspective of sexes in biological research.

## The genetic basis of complex phenotypes

Polymorphisms often involve multiple co-expressed or correlated traits. A major question in the study of polymorphism is how this occurs at the genetic level, because genetic recombination can break apart gene complexes even if their co-expression is adaptive (Schwander et al. 2014; Llaurens et al. 2017). Sexes represent a similar challenge. How does the association of traits like gonads and color differences occur and persist when individuals are perpetually recombining their genomes? The pathways to trait associations are numerous but often occur through either a mechanism that reduces genetic recombination, or changes to a shared regulatory mechanism with pleiotropic effects such as a hormone or transcription factor.

**Fig. 1:**
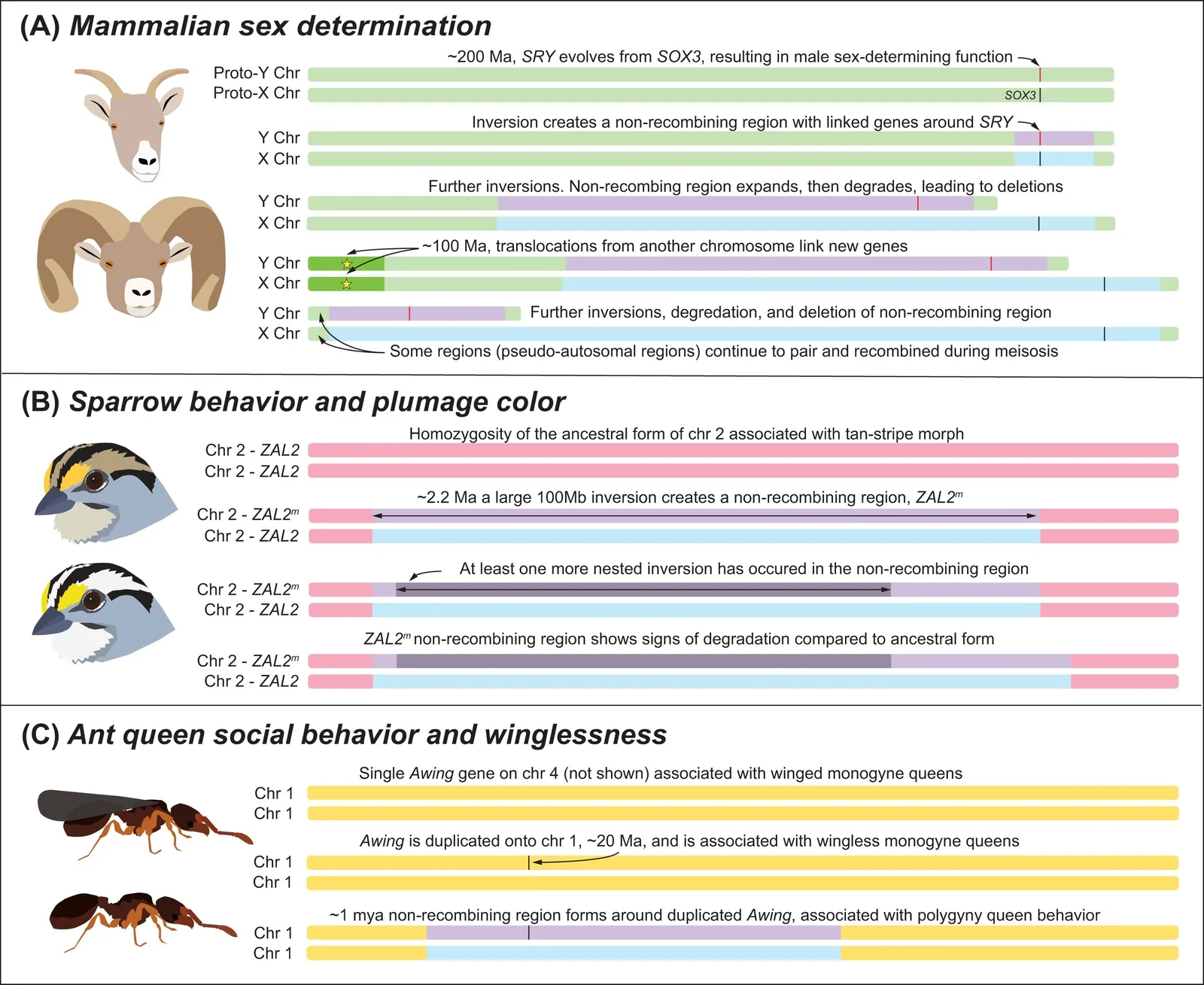
Examples of supergene evolution. **(A–C)** Each progressive pair of chromosomes shows the hypothesized supergene evolution from ancestral to modern forms. Purple denotes non-recombining chromosome regions. Blue denotes regions ancestrally homologous to the non-recombining region but that continue to recombine during homologous pairings. Each panel shows a simplified model, not drawn to scale and not definitive. **(A)** The mammalian sex chromosomes are former autosomes that gained a sex-determining function some 160-200 Ma when *SRY* evolved from *SOX3*. A series of inversion mutations around *SRY*, driven by genetic sexual antagonism, led to a non-recombining region on the Y chromosome with high linkage to *SRY*. Roughly 100 Ma, a translocation from an autosome likely brought more genes to the sex chromosomes. Non-recombining regions tend to degrade over time due to accumulated mutations and purging maladaptive alleles, eventually leading to the highly heteromorphic X and Y seen in humans. Like other supergenes, some regions on the Y continue to retain ancestral recombination capabilities with X (pseudo-autosomal regions). Based on Fig. 3 from Lahn et al. (2001). Rocky Mountain bighorn sheep, *Ovis canadensis canadensis*, are shown as an example. **(B)** Like sex chromosomes, non-sex polymorphisms can evolve through structural mutations. A supergene spanning a large portion of chromosome 2 in the white-throated sparrow (*Zonotrichia albicollis*) dictates a plumage-color and behavioral polymorphism. The tan-striped morph develops from the ancestral chromosome. Roughly 2 Ma, a large ~100 Mb inversion created the supergene, and there have likely been nested inversions within the gene since then (Thomas et al. 2008; Jeong et al. 2022). The inversion links over a thousand genes, many of them thought to influence behavior (Merritt et al. 2020). There is degradation on the non-recombining region, much like what is seen in the evolution of heteromorphic sex chromosomes but to a lesser degree. **(C)** The genetic antagonism that drives sex chromosome evolution can also be found in non-sex polymorphisms*. Myrmecina graminicola* ants have queens that are polymorphic (Mona et al. 2025). Monogyne queens are typically winged before colony formation and disperse from their natal colonies to singly create new colonies. Polygyne queens are wingless and form new colonies with other queens and their workers. The *Awing* gene is ancestrally on chromosome 4, which produces the monogyne type. Roughly ~20 Ma, a duplication of *Awing* onto chromosome 1 caused wingless monogynes. More recently, a ~20 Mb region around the duplicated *Awing* caused the evolution of the wingless polygyne queen. Researchers hypothesize that wings are only beneficial to monogyne queens due to their dispersal behavior, but wasteful to polygyne queens, which do not disperse (Mona et al. 2025). Such genetic “social antagonism” is akin to sexual antagonism in that reduced recombination through supergene formation is favored when traits are beneficial to one morph but not the other.

Perhaps the most studied type of such phenomena is the heteromorphic sex chromosome, for example, the X and Y (Fig. 1A) or Z and W chromosomes, in which paired sex chromosomes are morphologically distinguishable. The “classic” model of sex chromosome evolution often taught in biology courses begins when an autosomal gene evolves a sex-determining function (Charlesworth and Charlesworth 1978; Charlesworth 1991). Once this occurs, there may be phenotypic traits that are advantageous in one sex but not the other (sexual antagonism), so there is selection for reduced recombination of traits around the sex-determining gene (Rice 1984). Reduced recombination can occur most dramatically through large inversion mutations, but also through other structural mutations like insertions or deletions (Lahn and Page 1999). Over time, recombination can become so thoroughly restricted on one of the chromosomes that the only pathway to purge maladaptive mutations is through deletion, potentially leading to highly diverged sex chromosomes. It should be noted that exceptions to this model are remarkably commonplace, and it is unlikely that sex chromosome evolution follows this linear path in most cases (Bachtrog et al. 2014; Furman et al. 2020; Renner and Müller 2021). Key assumptions, like whether sexual antagonism is the main driver of recombination suppression, are difficult to test, and alternatives have been suggested (Ironside 2010; Wright et al. 2016; Ponnikas et al. 2018). Nevertheless, a few key traits remain in many cases of genetic sex determination—(1) one sex carries two similar chromosomes, while the other carries two different ones, (2) structural mutations suppress recombination around a sex-determining gene or genes, and (3) some degree of degeneration in the region with reduced recombination on the sex-limited chromosome.

Box 2 Glossary.

**Polymorphism**—the occurrence together in the same habitat of two or more distinct forms (i.e., *morphs*) of a species (see Box 1; Ford 1945).

**Polyphenism**—a type of polymorphism in which environmental conditions trigger development into one or another morph.

**Reaction norm (norm of reaction)**—the pattern or range of phenotypes that result from a single genotype across a range of environments.

**Gonochoristic**—each individual produces only microgametes or only macrogametes (Warkentin et al. 2026a).

**Hermaphroditic**—each individual produces both microgametes and macrogametes (Warkentin et al. 2026a).

**Parthenogenesis**—a form of asexual reproduction in which a gamete develops into a new individual without undergoing syngamy.

**Heterodichogamy**—a polymorphism where individuals reciprocally vary in temporal male or female function, with some individuals operating as male-first/female-second, while other individuals are female-first/male-second.

**Distyly**—a polymorphism where individuals reciprocally vary in the spatial separation of male and female reproductive structures. Individuals are either “pin” (long style/short stamen) or “thrum” (short style/long stamen).

Non-sex polymorphisms (a polymorphism that does not confer size-differentiated classes of gametes) often follow this same pattern. One example is a complex polymorphism in the white-throated sparrow, *Zonotrichia albicollis*. In this species, individuals express either a “tan-stripe” or “white-stripe” color polymorphism that is also linked with differences in mating and parental behavior (Tuttle 2003). There is strong disassortative mating such that each morph almost always prefers to mate with the opposite type (Tuttle et al. 2016). Studies have shown that the genetic evolution behind these two morphs is remarkably similar to a sex chromosome (Tuttle et al. 2016). The polymorphism in this case arises from an autosomal supergene—that is, several physically linked genes that are inherited together—referred to as *ZAL2^m^*. The *ZAL2^m^* supergene is massive, taking up almost the entire length of the chromosome, and contains over a thousand genes (Fig. 1B; Tuttle et al. 2016). Like the mammalian Y chromosome, *ZAL2^m^*’s modern form arose from multiple large inversion mutations on an autosome that have led to a region of suppressed recombination (Fig. 1B; Thomas et al. 2008). Studies have found signs of degeneration, such as accumulation of non-synonymous mutations, reduced expression of *ZAL2^m^* genes, and lower genetic diversity (Tuttle et al. 2016; Sun et al. 2018; Jeong et al. 2022). Interestingly, though signs of degradation are present, they also appear to be weak, potentially due to recombination in rare *ZAL2^m^* homozygotes (Jeong et al. 2022). Rare recombination events have also been hypothesized to play a role in reducing degradation in sex chromosomes (e.g., Perrin 2009).

Another example comes from a polymorphism in ants, in which colonies can be either monogyne (one queen) or polygyne (multiple queens) (Kay et al. 2022). Monogyne colonies reproduce when a single reproductive female establishes a new colony far from her mother colony and are intolerant of ants from other colonies. Polygyne colonies reproduce instead by budding to form nearby colonies that are tolerant of multiple queens and their workers. In the fire ant *Solenopsis invicta*, a supergene underlies this polymorphism (Wang et al. 2013). Monogyne queens are homozygous for the ancestral form, while polygyne queens are heterozygous with one copy of the *Sb* supergene that spans a major portion of an autosome (Wang et al. 2013). Here again, there is evidence that *Sb* is undergoing degeneration through accumulation of repeated elements and low diversity (Pracana et al. 2017; Stolle et al. 2019). There is also evidence in *Formica* ants that the supergene related to this social polymorphism is under selection for reduced recombination due to “social antagonism” wherein queen miniaturization is only beneficial to polygyne colonies (Scarparo et al. 2023). Remarkably, the supergene that regulates this polymorphism has an expanded region of reduced recombination that includes genes on another chromosome, which is hypothesized as a way to reduce recombination between queen miniaturization and polygyne phenotypes (Scarparo et al. 2023). This type of genomic conflict is often discussed in terms of sexual conflict (i.e., sexual antagonism), when certain alleles are advantageous for one sex but not the other (Bonduriansky and Chenoweth 2009). To reduce such conflict, sex chromosomes can fuse with autosomal elements to reduce recombination (Fig. 1A; Charlesworth et al. 2005), analogous to the above example in *Formica* ants. While genomic conflict is most often discussed in terms of sexes, conflict between other types of within-species polymorphism could select for similar genomic solutions (see Fig. 1C for a related example of social antagonism in ants). While many of the examples in this paper suggest ways in which the study of sex can be informed by research on polymorphisms, this example shows how a large body of research on sexes can inform research on the evolution of polymorphisms.

The examples above demonstrate how heteromorphic sex chromosomes are a type of supergene, with little distinction besides sex chromosomes being defined around their relationship to gametic size classes (Schwander et al. 2014; Black and Shuker 2019). However, supergenes are far from the only way that the expression of complex phenotypes can be regulated. For example, a female color pattern polymorphism in brown anoles (*Anolis sagrei*) is associated with a single gene region, but is adjacent to an estrogen receptor gene (Feiner et al. 2022). The two genes are co-expressed in developing embryos with lower overall expression in males, which explains why only females express the phenotypic variation (Feiner et al. 2022). Sex determination can also involve remarkably small regions of the genome, resulting in homomorphic sex chromosomes that are barely differentiated. Sexual phenotype in the tiger pufferfish (*Takifugu rubripes*) is regulated by a single nucleotide polymorphism that changes an amino acid of the *Amhr2* gene, and there are no signs of recombination suppression in the sex chromosome (Kamiya et al. 2012). Date-plum persimmons (*Diospyros lotus*) have genetically determined sex, and in this species, the only sex-differentiated region of the genome is the *OGI* locus, which does not encode a gene, but rather a small 21-basepair RNA structure that blocks the expression of *MeGI*, an autosomal gene that causes the expression of female flowers (Akagi et al. 2014, 2016). Complex variation can arise from endless genetic architectures, and it should come as no surprise that sexes reflect this same diversity (Kocher et al. 2024).

## Plasticity, environmental effects, and reaction norms

Of course, sex is not always genetically determined. Environmental sex determination has been studied extensively across taxa (Bachtrog et al. 2014; Capel 2017; Pannell 2017). Temperature- and social-dependence are two of the most well-known types. In the red-eared slider turtle, for example, lower temperatures increase the expression of *Kdm6b*, which then leads to increased demethylation of *Dmrt1*, resulting in the male phenotype (Ge et al. 2018). In a fern, *Ceratopteris richardii*, haploid spores develop into hermaphrodite, female, or male gametophytes depending on the social environment (Tanurdzic and Banks 2004; Pannell 2017). Hermaphrodites secrete a pheromone, antheridiogen, which causes the expression of the *HER* gene, which ultimately leads to the development of males. In the absence of antheridiogen, *HER* is not expressed, leading to development of females or hermaphrodites (Tanurdzic and Banks 2004; Pannell 2017). Sex plasticity, or changing from one sex to another, is an especially notable form of environmental sex determination that plays an important role in many plants and animals (Munday et al. 2006; Vega‐Frutis et al. 2014).

Environmentally induced morphotypes can also be found outside the context of sexes. Such *polyphenisms* are a type of phenotypic polymorphism (Box 1). As with environmental sex determination, certain environmental or social cues can cause an individual to develop into one of multiple distinct morphotypes. For example, the development of the reproductive queens in honeybee nests through feeding of “royal jelly” is one well-known example (Weaver 1955). In some dung beetles, males will either grow a short or a long horn, depending on their diet during larval development (Moczek 1998). In another classic example, spadefoot toad tadpoles have a polymorphism that dictates size and morphology and is influenced by diet (Pfennig 1990). The omnivore morph is smaller, with a more rounded head and smaller jaws, while the carnivore morph has a larger body size and jaw musculature, shorter gut, and distinct teeth morphology (Levis et al. 2018). Researchers have proposed a variety of cues that appear to lead to the development of the carnivore morph, such as higher availability of high-protein foods like fairy shrimp (Pfennig 1990), or increased absorption of thyroxine hormone (Pfennig 1992).

Though plastic, it has long been understood that the degree of plasticity in spadefoot tadpole morphotypes is heritable (Newman 1988), and recent research shows that even the morphotype is also heritable to some degree (Isdaner et al. 2024). Different populations differ in their propensity to become one morph or another under the same environmental conditions (Isdaner et al. 2024). In other words, while these morphs are highly influenced by the environment, some individuals are more likely to develop the carnivore phenotype. This should come as no surprise, because phenotypic variation is comprised of variation due to genetic variation, environmental variation, and their interaction (*V_P_ = V_G_ + V_E_ + V_GE_*).

Again, this is true for sex determination as well. Turtles are often cited as an example of temperature-dependent sex determination, as described above, but even here, populations show heritable variation in the threshold temperature at which different sexes develop (Janzen 1992; McGaugh et al. 2011). Sex is determined by clear genotype by environment interactions in many other species as well. For example, in the three-lined skink (*Bassiana duperreyi*), phenotypic sex is regulated genetically in an XY system, but at temperatures below 20 Celsius, all individuals develop male reproductive organs (Shine et al. 2002). In the previous section, I described genetic sex determination in date-plum persimmon. However, in a closely related polyploid persimmon, methylation of the *MeGI* gene allows for the expression of female flowers on "genetically male" trees (Akagi et al. 2016). Even in systems considered to be genetic in their sex determination, “sex reversals” occur in which there are XX males or XY females, especially in amphibians and fishes (Perrin 2009). In many of these cases, sex-reversed individuals are fully fertile (Perrin 2009). X and Y chromosomes appear to readily recombine in phenotypic females, an unexpected finding given that structural mutations are typically assumed to be the causative mechanism that prevents recombination between heteromorphic sex chromosomes. By creating an opportunity for recombination, sex reversals have profound evolutionary effects by preventing degradation of the Y chromosome, even when highly rare (Perrin 2009). Without deliberate confirmation of a “match” between phenotypic and genetic sex, sex reversals such as these can be difficult to detect, but may have important evolutionary effects.

**Fig. 2:**
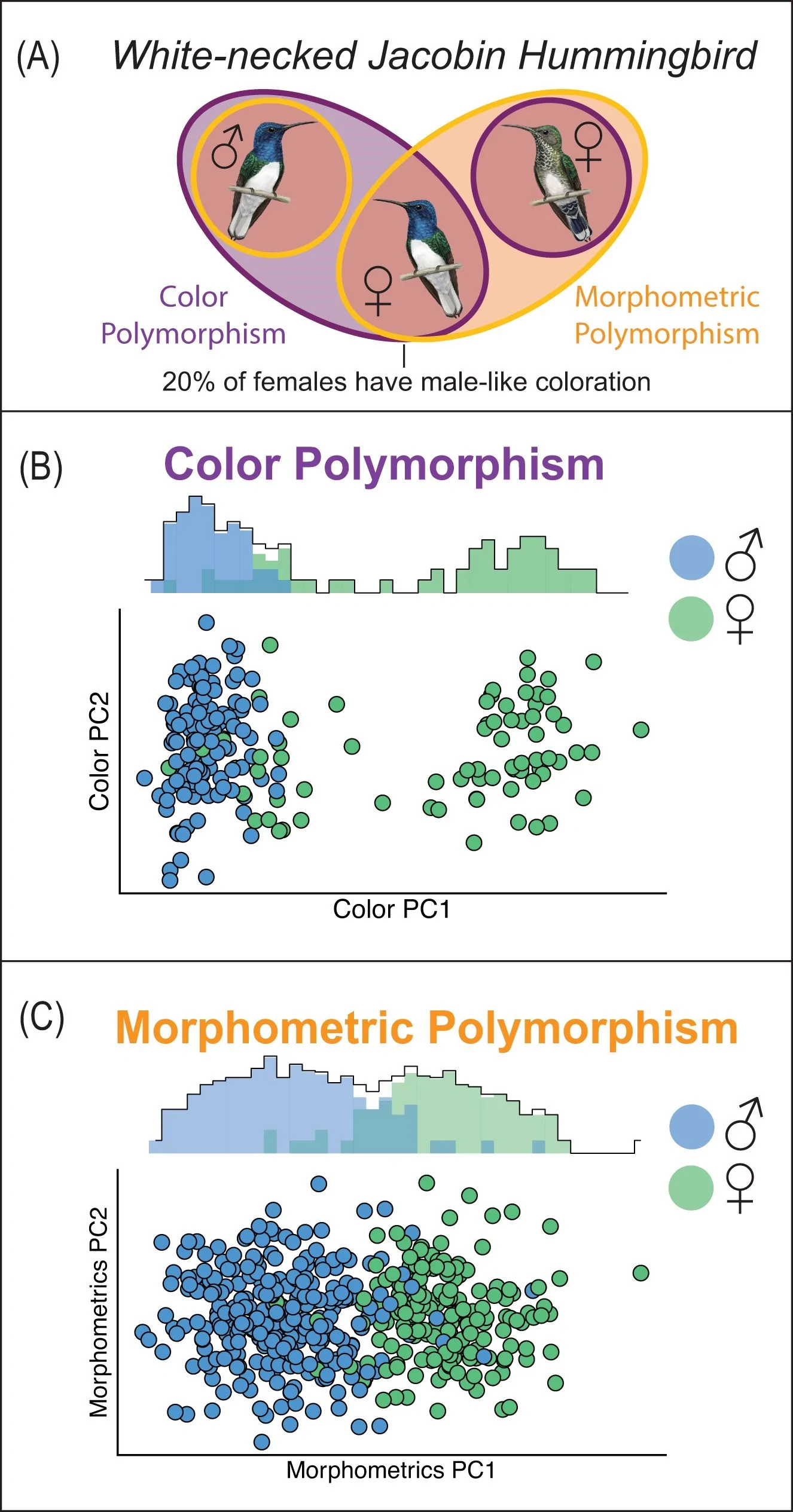
Describing layers of polymorphic variation. **(A)** The white-necked jacobin hummingbird (*Florisuga mellivora*) is typically described as having a female-limited polymorphism: about 20% of adult females have male-like coloration, while the other 80% are distinct from males. The same variation can instead be described as layered polymorphisms—one polymorphism in a set of linked plumage-color metrics, and another in linked morphological traits. Both are linked to genetic sex, but in different ways. **(B, C)** The first two axes of principal component analyses of coloration or morphometrics, with blue and green indicating genetic males and females, respectively. A histogram of PC1 (log-transformed) is included, plotted separately for each sex, with a dark line showing both sexes combined. **(B)** Coloration falls into two distinct modes with few intermediate individuals. Males occupy a single mode, whereas females occupy both. The color polymorphism is therefore relatively discrete, but genetic sex maps onto only one of the two modes. **(C)** Morphometric measures show two clusters and are therefore polymorphic (see Box 1). Each sex predominates a different peak, but there is considerable overlap in the tails. Further research may show additional layers of variation, some covarying with color, others with morphology and sex, and some independent of both. Genetic sex could be viewed as a third polymorphism that is relatively discrete and bimodal. Data from Falk et al. (2021, 2022). Hummingbird illustrations by Jillian Ditner.

The interaction between genotype and environment is formalized under the concept of *reaction norms*—the range of phenotypes produced by certain genotypes under varying environmental conditions. While reaction norms are frequently discussed in environmental sex determination, the concept is rarely invoked in species with so-called “genetic” sex determination even though environmental factors can and do have effects in such systems (Ah-King and Nylin 2010; Kocher et al. 2024). This is unfortunate, because it casts intersex conditions to the realm of anomaly (Ah-King and Nylin 2010) when they should be expected, given that all traits have reaction norms. They can also have profound evolutionary impacts, as with the previously mentioned sex-reversed amphibians. With reaction norms, occasional or rare deviations from expectation based on genetic sex (i.e., “sex reversals”) are not necessarily an oddity, but part of a very real and potentially important developmental pathway that varies under different environmental conditions. For example, it has long been known, and documented even by Aristotle, that female chickens will readily express “rooster” traits like wattles and long spurs (Forbes 1947). Moreover, though all birds are considered to have genetic sex determination with heteromorphic sex chromosomes, a recent study across 5 distantly related bird species found that chromosomal sex did not completely match the gonadal sex in 3–6% of individuals (Hall et al. 2025). Furthermore, 20% of these “sex-discordant” individuals may still be able to reproduce since their gonads showed no morphological indication of reduced functionality (Hall et al. 2025). The taxonomic distance between these 5 species makes it difficult to cast aside these result as anomaly, yet the evolutionary implications of this phenomenon have not been studied. Furthermore, the use of genetic sexing is widespread in biological research with an implicit assumption that chromosomal sex is entirely congruous with gonadal and gametic sex (including in the author’s own work, Fig. 2). Though it remains to be seen whether this sex discordance is a widespread phenomenon, it should certainly raise doubt on the strictness of the correlations between chromosomal, gametic, and gonadal phenotypes. Surprising findings such as this should perhaps not be a surprise. As with all forms of biological variation, there are always exceptions. By assuming that sexes follow a different set of rules than other types of variation, we risk overlooking important and fascinating biological phenomena.

## The persistence and disappearance of phenotypic variants

How can multiple morphs persist when directional selection and genetic drift tend to reduce variation over time? In some cases, the existence of multiple variants within a population is transient, and there is active transition toward the fixation of the adaptive morph (Ford 1945). Repeated or consistent mutation and migration between populations can also cause persistence of non-adaptive variation. However, certain types of selection can also encourage the persistence of morphs through negative frequency-dependent fitness, heterogeneous environments, or genetic overdominance (Hedrick 2007).

Negative frequency-dependence can arise through social and ecological interactions, giving rare morphs an advantage. For example, apostatic selection occurs when predators detect their prey through a “search image” (Bond 2007). Prey with a common appearance are easier to detect, and will have lower fitness, but a rare morph with a novel appearance could escape predation and have relatively higher fitness, and subsequently increase in frequency in the population. At the same time, however, predators will develop a new search image for the novel form, leading to decreased fitness. This oscillation in fitness prevents the fixation of any morph. Similar dynamics occur in scale-eating cichlids, which have asymmetric mouths that aid in attacking other fish from the side (Hori 1993). “Left” morphs are better at attacking prey on their right sides, and vice versa. Certain types of mimicry can also result in the persistence of multiple variants. For example, in the white-necked jacobin hummingbird (*Florisuga mellivora*), females have a color polymorphism—most are distinct from males, but roughly 20% of females are nearly identical (Fig. 2; Falk et al. 2021). Males are generally more aggressive than females, deterring other hummingbirds from trying to chase them away while feeding from nectar resources (Falk et al. 2021, 2022). By mimicking males, females are able to obtain these same benefits, allowing them to feed more efficiently without actually being any more aggressive (Falk et al. 2021). When male-mimicking females are common, they tend to get attacked more, creating an equilibrium where neither morph is likely to overtake the other (Falk et al. 2022, 2025).

Are external selective forces like balancing selection necessary to maintain both sexes? After all, are not both sexes necessary to reproduce? Indeed, sexes have been described as functionally dependent morphs because gonochorist males and females cannot reproduce without each other (in contrast to functionally independent morphs, like the two female hummingbird types, which do not depend on each other) (West-Eberhard 2003b). However, there are other functionally dependent morphs in nature, such as the queens and workers of a bee or ant colony, so mutual dependence is not a unique trait of sexes and cannot alone explain why they persist. In fact, it is the same types of mechanisms of balancing selection discussed previously—negative frequency-dependent selection, heterogeneous environments, and overdominance—that maintain sexes as well.

Negative frequency dependence plays an especially prominent role in the balancing of sex ratios. In species where every individual has one mother and one father, the total reproductive success of females and males must be the same. This simple accounting constraint is sometimes called the “Fisher condition” (Fisher 1930), and it holds whenever offspring are produced biparentally from two distinct sexes (rather than, e.g., species where individuals can have only one parent, such as in species with haplodiploid sex determination or in parthenogenetic reproduction). When sexes are equally common, total fitness of all individuals is divided among equal numbers of males and females, so the mean fitness of males and females is the same. Now, suppose females are rare: the total fitness of all males and females still must be equal, but it is divided between fewer females than males, so each female’s mean contribution exceeds each male’s mean contribution. Under such conditions, individuals with more female offspring will have higher fitness, and if this tendency is heritable, the number of females should increase until females are more common than males. The Fisher condition therefore creates strong negative frequency-dependent selection for a roughly equal sex ratio. Notably, no sex-specific features of being female or male are necessary for the process described above—only biparental reproduction from two different types (e.g., distyly, heterodichogamy, or the white-throated sparrow polymorphism), yet it is almost exclusively discussed in terms of males and females. The Fisher condition also plays an important role in models of parental care and mating systems, because matings must balance between the sexes. Some early models of parental care failed to account for the Fisher condition (e.g., Smith 1977), leading to an overly simplified explanation for the connection between gamete size and parental care that went unnoticed for decades (Kokko and Jennions 2008; Fromhage and Jennions 2016). Had modelers reasoned from the outset that mating must be balanced between the sexes, they might have avoided assumptions that still fuel misconceptions today.

Negative frequency-dependent selection can maintain polymorphisms across remarkably long periods of time, but can eventually give way to strong directional selection or drift, resulting in a single morph. In a similar way, asexual or hermaphroditic lineages with no sexual differentiation can arise from species with balanced sexes. For example, balanced sexes can give way to populations of *parthenogenic* individuals. This appears to be very common in phasmid stick insects, with roughly 39% of studied species demonstrating either facultative or obligate parthenogenesis (Schwander et al. 2026). Though many species are facultatively parthenogenic, most natural populations exhibit high rates of either sexual or parthenogenic strategies (Schwander et al. 2026), indicating that under certain circumstances the benefits of parthenogenesis outweigh the benefits of sexual reproduction. Some species that are comprised purely of obligate parthenogenic females have been observed to occasionally produce reproductive males through the loss of the X chromosome, providing a pathway to regaining sexual reproduction, which is thought to have occurred in *Clitarchus hookeri* (Morgan-Richards et al. 2019). The evolutionary alternation between balanced sexes and hermaphrodites is also extremely common, especially in plants (e.g., Goldberg et al. 2017).

One of the reasons that sexes appear to be so fundamental is that the long-term maintenance of polymorphism may reach extremes when it comes to sexes. In mammals, the *SRY* gene has retained a sex-determining function for 180 million years, predating the placental and marsupial split (Cortez et al. 2014). Furthermore, though the specific sex-determining system varies considerably across taxa, the basic developmental pathway of gonadal differentiation is conserved across vertebrates (Capel 2017; Nagahama et al. 2021), demonstrating sex-differentiating elements that have been maintained across deeply diverged branches of the vertebrate phylogeny. The deep homology of sexual differentiation is a remarkable testament to the strength of selection to maintain sexual diversity. However, other types of polymorphisms also have remarkable staying power that extends across taxa. Plants have evolved a variety of mechanisms to promote self-incompatibility and reduce inbreeding, including gametophytic self-incompatibility in which pollen will only be accepted by a plant with a non-matching allele at an *S*-locus. In the Solanum family, similar *S*-locus alleles can be found across the family, indicating that the polymorphism existed before diversification of this speciose family more than 30 million years ago, and still exists to this day (Ioerger et al. 1990). The same *S*-locus mechanism for self-incompatibility is shared with other families with a common ancestor from more than 90 million years ago (Nowak et al. 2011). In the Primula family, individuals are polymorphic for style length, a trait known as heterostyly. This trait is linked to a supergene that arose following a duplication event that likely occurred 51.7 million years ago and is now shared throughout the family (Li et al. 2016). All of these polymorphisms, including sexes, represent a fascinating class of trans-species polymorphism in which selection to maintain diversity extends beyond speciation events.

## A polymorphic perspective of sexes: sexes are variants, sexes are variable, and some variants are “sex-like”

In the sections above, I show from a variety of perspectives how sexes are a type of morphic variation, just like many other types of morphic variation that we very often observe within species. A “polymorphism perspective” of sex is therefore not novel in the sense that it departs from any previously established framework. Rather, it is simply a shift in perspective from sexes as *entities* to sexes as *variation*. Such a shift can change how we view organisms, our assumptions about them, and ultimately how we ask questions during the scientific process.

If sexes are treated as a type of variation, classic analytical frameworks for studying phenotypic evolution can also be applied to sexes. I discussed one such example previously, in the concept of reaction norms. Another is the **G** matrix, which shows how a set of traits vary across individuals and how they tend to shift together. This allows researchers to predict direction of evolution in complex phenotypes given the type of selection and built-in constraints between traits (Lande 1982). Notably, this incorporates the “multivariate” aspect of sexual phenotypes described in the McLaughlin et al. (2023) model of sex. The related **B** matrix describes the covariation in traits between groups of individuals such as different sexes (Wyman et al. 2013; Houle and Cheng 2021), allowing for predictions of how sexual traits can evolve in terms of the independence of traits between sexes. These analytical frameworks help to consider sexes as having as much dynamism, evolvability, and responsiveness to both environmental and genetic variation as any other type of phenotypic variation (West-Eberhard 2003a; Anderson and Falk 2023).

A second advantage to the polymorphism perspective of sexes is that it contextualizes them as just one type of variation among a sea of other “layers” of variation, some related to reproduction and others not (Fig. 2). This is a major point against policies that mandate or strongly encourage the measurement of sex differences in all variables (e.g., “Sex as a Biological Variable” policy from the US National Institute of Health). DiMarco et al. (2022) argue that these mandates are too blunt of a tool to bring about improved outcomes through precision medicine because sex is only sometimes medically relevant. Instead, they propose a sex contextualism framework in which “sex related variables are not omnirelevant, but emerge as relevant or not within the context of a research program or other pragmatic aim.” While DiMarco et al. (2022) are primarily concerned with medical research and implications for humans, these ideas are highly applicable to organismal biologists. Sex is indeed a biological variable, but it is far from the only one, and sex itself may not be a relevant variable requiring attention, depending on the context of the questions we are asking (Massa 2026).

Another advantage of viewing sexes as variation is that it encourages greater precision and specificity. If sexes are not fundamentally different in their genetics, development, or evolution from other types of variation, it follows that sexes differ from other types of variants *only* in the way that we choose to define them. In other words, instead of debating what can and cannot be called “biological sex,” treating sex as variation puts emphasis on the definition itself, the way it is formulated, as well as the fact that defining sex in certain ways is itself a choice (Ritz and DuBois 2025). As other papers in this special issue have detailed, the term “sex” can have a variety of different meanings (Warkentin et al. 2026b). One way to appreciate the consequences of this conceptual flexibility is to consider organisms that push the limits of binary frameworks.

For example, there has been discussion about whether the white-throated sparrow can be described as having four sexes (Campagna 2016; Maney et al. 2020). Under a gametic definition of sex, there are two sexes associated with either sperm or egg production during adulthood. However, as we have previously discussed, the white-stripe/tan-stripe polymorphism is remarkably sex-like: Its genetic basis traces to a supergene that came to be through a series of inversions, leaving one haplotype under a state of reduced recombination and degeneration, much like a heteromorphic sex chromosome (Fig. 1B). The result causes a suite of dimorphic traits, from coloration to hormones and behavior. Like sexes, these are functionally dependent alternative morphs, because each relies on the other for reproduction. Note also that because almost all individuals are the product of a tan-morph and a white-morph, the system follows something that looks almost exactly like the Fisher condition, which causes strong negative frequency-dependent selection and a morph ratio of roughly 50/50.

Under a polymorphism perspective of sex, whether there are two or four sexes is somewhat beside the point, because at a basic level, we can definitively say that the white-throated sparrow has two independent, layered polymorphisms. At this point, we can define the two: the first is a gamete-associated polymorphism that restricts pairs based on gametic incompatibilities, and the second is a color-associated polymorphism that restricts sexual reproduction through a genetic incompatibility. Functionally, both the color polymorphism and the gamete-associated sexes do the same thing—they restrict and facilitate which individuals in the population are reproductively compatible.

Other polymorphisms can also play highly sex-like roles. For example, heterodichogamy is a form of sequential hermaphroditism found in flowering plants where individuals have a temporal separation between female and male function. A polymorphism dictates whether individuals are male-first/female-second or female-first/male-second. The change can happen over the course of a flowering season, such as in walnuts, or the cycle can repeat on a daily basis, such as in many avocado species. In pecans, recent work on the genetic basis of heterodichogamy shows the involvement of a supergene spanning about 20 genes (Groh et al. 2025). Here again, we see evidence for reduced recombination and degradation in the form of accumulated repetitive elements. The supergene variant is likely over 50 million years old, on par with the age of some heteromorphic sex chromosomes (Zhou et al. 2013). All individuals of heterodichogamous species are hermaphroditic, so in this case there is no polymorphism associated with gamete type. Far more than the actual “sex” of these individuals, the heterodichogamy polymorphism dictates who is able to mate with whom. A similar scenario occurs in distylous plants, in which a reciprocal polymorphism dictates whether female- or male-functioning organs are spatially more accessible to pollinators. Again, distylous plants are hermaphrodites, but in this case outcrossing is regulated by spatial separation, rather than temporal separation, or gamete fusion compatibility.

We can call these polymorphisms “sex-like,” but another perspective is that all of these polymorphisms are involved in restricting and facilitating possible mate pairs, with gametic restrictions as just one of several possibilities. In some organisms like the white-throated sparrow, both gametic and non-gametic variants play critical roles in determining sexually reproductive pairs. In others, such as pecans, gametes play no role while timing incompatibilities play a much larger role. Gamete incompatibilities are in fact several types of incompatibility layered together, many of which are not related to size classes. Phenotypic polymorphisms can play almost no role, for example, in basidiomycete fungi, wherein incompatible mating types are only distinguishable at the genetic level, not necessarily by phenotype. Other non-gametic phenotypes that play important roles in restricting and facilitating mates include mate preferences and pollinator syndromes. The functional role of sexes as gamete-associated traits is not unique and exists within a multitude of similar mechanisms that play the same evolutionary and reproductive role.

As a final point, it is important to note that a polymorphism perspective cannot adequately describe sexual function for all sexually reproducing organisms. For example, all individuals of hermaphroditic species are not split perfectly between their sexual functions. Instead, as with most traits, there is inevitable variation. In the context of sexual reproduction, hermaphroditic individuals can vary in the relative production of different gamete size classes, and in individuals contribution to the next generation made through these gametes (Lloyd 1980; Pannell 2023; Casper 2026; Casper and Warkentin 2026). While in some cases the distribution of sexual function may be polymorphic (e.g., Saumitou-Laprade et al. 2018), in perhaps most hermaphroditic species it is more accurately described as continuous. There is still great utility in viewing sexes as within-species variation, even when that variation is not polymorphic.

## Conclusion

Describing sexes within the broader landscape of biological variation restores conceptual continuity between sex and other phenotypes, granting it the same analytical tools and conceptual frameworks that have been productive across evolutionary and organismal biology. Sexes can be understood as components of a variable, evolvable phenotype—one in which gametic variation is potentially important but not necessarily foundational. The result is a framework that accommodates biological diversity without forcing it into binary categories, enables more precise comparative inference across taxa, and positions sex as a natural subject of the same evolutionary and organismal questions we ask of variation more broadly. Sex is often treated as a fundamental state of being that dictates nearly every aspect of life, and it can be easy to slip into such assumptions when it comes to organismal research. Adopting a polymorphism perspective of sexes does not necessarily mean the adoption of specific new practices. Rather, this is a subtle shift in perspective that takes what is often treated as special, and grounds it to a more accurate placement among all other types of variation. At minimum, it is a more accurate representation of biological reality; at best, it reduces hidden assumptions, opens new comparative questions, and generates novel ways to understand the diversity of sexual phenotypes across the tree of life.

## Data Availability

No new data were created or analyzed in the preparation of this manuscript.

## Notes

From the symposium “Sex across origins: Questioning animal-centric assumptions and developing integrative frameworks” presented at the annual meeting of the Society for Integrative and Comparative Biology, January 3–7th , 2026.
